# Psychological complications of the children exposed to domestic violence: a systematic review

**DOI:** 10.1186/s41935-023-00343-4

**Published:** 2023-05-26

**Authors:** Alireza Doroudchi, Mohammad Zarenezhad, Homayoun Hosseininezhad, Abdorrasoul Malekpour, Zahra Ehsaei, Reza Kaboodkhani, Maryam Valiei

**Affiliations:** 1 Legal Medicine Research Center, Legal Medicine Organization, Tehran, Iran; 2 Department of Otolaryngology and Head and Neck Surgery, Shiraz University of Medical Sciences, Shiraz, Iran

**Keywords:** Domestic violence, Child, Psychology

## Abstract

**Background:**

Domestic violence (DV) is one of the major concerning health problems worldwide, and individuals who experienced domestic violence, may suffer physical or psychological consequences.

**Main body:**

Children as a major part of the family and society are usually involved in familial challenging events such as domestic violence, and suffer several major complications. This review aimed to explore the psychological consequences of child exposure to domestic violence. A systematic search in four databases yielded 18 peer-reviewed original studies that met the inclusion criteria. Selected studies highlighted the psychological consequences of domestic violence in witnessing and exposed children. The findings of the present study revealed that children exposed to DV suffer various psychological, mental, and behavioral complications which may be short and long-lasting, and moderate or severe. Children exposed to domestic violence may show declined educational performance and social abilities. Internalization problems, depression, post-traumatic stress disorders symptoms, and externalization symptoms such as aggressive behaviors and even lower levels of IQ are of most important reported complications of domestic violence in children. Educational programs for parents as well as trained school caregivers and health policy-makers can diminish and prevent the complications of domestic violence in children.

**Conclusion:**

Considering the importance of children mental hygiene, health policymakers should consider facilities to screen and detect children with signs of maltreatment and exposed to domestic violence. In this regard, trained teachers or school counselors will be helpful, as social supports and therapies may be more effective by early detection affairs.

## Background

Domestic violence (DV) as a major worldwide health problem, has attracted the attention of social and health policymakers (Finley 2019, Marchetti 2019, Shayan et al. 2015). Family (domestic) violence lead to physical and psychological consequences affecting all family members, even relatives (Ferrari et al. 2016, Noble‐Carr et al. 2019). DV includes various types of violence such as physical, sexual, and emotional; mainly referred to intimate partner violence, mostly occur between male and female partners. However, it can embrace child, elderly, or sibling abuse (Kourti et al. 2023). DV has been highlighted as a major health problem to children worldwide. For example, in the United States of America, about 3 million children have suffered DV consequences (Mastorakos and Scott 2019). Based on UNICEF reports, about 133–275 million children experience the exposure or witness DV (Unicef 2006). During COVID-19 pandemics, DV has also been highlighted as a critical and substantial public health issue (Wake and Kandula 2022). Repeated exposure to DV is believed to be associated with the onset, severity, and recurrence of mental health problems (Jouriles et al. 2018, Münger and Markström 2018, Zarenezhad et al. 2016) and individuals with mental disorders are at a higher risk of DV complications (Khalifeh and Dean 2010, Vaziri et al. 2018). DV endanger the health and quality of life of the family members including/especially the children (Hester et al. 2015, Ferrari et al. 2016, Knight and Hester 2016, Carneiro et al. 2017, Jaffe et al. 2017, David and Jaffe 2018). It is also believed that DV has more emotional and less physical manifestations (Callaghan et al. 2017, Ali and McGarry 2020). Children as a major part of family and society are usually indirect victims of DV and may suffer severe and irreversible physical and mental impacts from DV, and are at an increased risk for major mental health problems (Ferrari et al. 2016, Gomma et al. 2019, Hall 2019). However, psychological effects of DV on children have not been explored systematically. Thus, the present systematic review aims to demonstrate the psychological impacts of DV on children.

## Methodology

Medical subject headings were used to search related published studies in scientific databases including PubMed/MEDLINE, Thomson Reuters, Cochrane, and Google scholar. The search was conducted in the mentioned databases using Mesh keywords Domestic violence, Children (child), witness, exposed (Exposure), and psychological effects. Our search results included original research papers published in the English language from January 2000 until 2022, with their online abstracts. The study was conducted based on the inclusion criteria demonstrated in Table 1. Although our literature search was based on the Mesh keywords, studies with no mentioned key words but which covered the main topics and subject related to our subject were also included.

**Table 1:** Inclusion criteria for selection of the manuscripts in the systematic review

Criteria	Included	Excluded
Article type	Original Research Articles	Books, review articles, case reports, letter to editors, mini-reviews, published thesis
Language	English	Other languages
Descriptive/intervention	Descriptive studies	Interventional studies
Ethnicity	All nations worldwide	–
Publication time	From 2000 January until 2022 December	1999 and before
Keywords	Domestic violence, Children, Psychological impacts, exposure, Witness	–

### Data extraction and analysis

Data were extracted from the selected papers by two reviewers. Each article was evaluated separately and the results were merged. Discrepancies were resolved by referral back to the original papers. Extracted data included information about the authors of the studies, the year and country of the research, sample size, and the main findings of the studies. The results of the studies were merged in the discussion section considering other publications to respond to the questions: How do children suffer from domestic violence?

Do children suffer DV physically or psychologically?

What types of psychological disorders have children suffered because of domestic violence?

Does DV affect social activity or educational performance of the children?

Does DV affect Intelligence Quotient (IQ) or emotional intelligence (EQ) of the children?

Do children express delayed consequences of DV?

In our systematic review, the results were evaluated concerning the design and quality of the studies. The results of the studies were not combined due to the heterogeneity of the study types, populations, and outcomes. So, statistical analysis was not performed on the findings of selected studies.

## Results

Totally, 2740 potentially relevant articles were found in the first-round search in the mentioned databases. By screening titles of found articles; 2225 titles did not meet the criteria to be included in the study and were excluded. Five hundred fifteen articles potentially fulfilled the inclusion criteria and went forward for screening for duplicates in Endnote Software version X7. In this stage, 150 records were removed and 365 articles were evaluated by two reviewers independently concerning inclusion criteria. The abstract of selected papers was reviewed and papers were selected considering the subject and inclusion criteria of the study. In this stage, 284 papers were excluded and 81 articles were evaluated for full-text screening. Finally, 63 articles were excluded by screening the full-text body of articles and 18 articles were selected for the final systematic review. In the present systematic review, we included articles evaluating the psychological impacts of domestic violence on children that were published from 2000 to 2020.

Almost all studies indicated the negative impacts of domestic violence on children’s psychological status and their behavior. In the first study conducted by Levendosky and Bermann (2001), 120 women and their children residing in the community or domestic violence shelters were studied. Their findings indicated an ecological model for the impact of domestic violence on children and also the ecological framework and trauma theory exploring the effects of domestic violence on children.

Huth-Bocks et al. (2001) evaluated the direct and indirect effects of domestic violence on young children’s intellectual functioning. They included 100 women and their 3–5-year-old children (44 boys and 56 girls). They found that domestic violence has major impacts on verbal abilities and visual-spatial abilities by causing maternal depression and the intellectual quality of the home environment.

In another study, Levendosky et al. (2002) evaluated trauma symptoms in 62 preschool-age children exposed to domestic violence. The authors indicated that living with domestic violence is related to more aggressive behaviors and PTSD symptoms.

Children who witness DV may also suffer behavioral problems. For example, Spilsbury et al. (2008) studied 1019 children who were witnessing DV and they found that domestic violence caused symptoms of psychological maladjustment including externalizing and internalizing problems. Torteya et al. (2009) also reported that children exposed to DV showed significantly more internalizing or externalizing problems. They also found that chronic DV was associated with difficult child temperament and internalizing or externalizing symptoms.

Fusco and Fantuzzo (2009) evaluated the effects of direct exposure and the consequences of the involvement of DV on children. The children were involved as a part of the precipitating event, called for help. Almost, 75% of all children exposed to DV were directly involved in the violence, and were involved physically and mentally.

Rigterink et al. (2010) also evaluated the effects of DV on emotion regulation in children’s by measurement of vagal tone (VT). Almost, all children showed increased VT baseline, while DV-exposed children had less increase in baseline VT compared to non-exposed children. So, DV may sensitize children to stress and children continue to show increased stimulation to the stress, and their physiological resources become depleted as demonstrated by low baseline VT.

Fortin et al. (2011) conducted their study with 79 children exposed to DV. Indicators used for children’s appraisals of violence were attribution of blame and perceived threat. The levels of parentification and the degree of children’s faithfulness conflicts were assessed for children’s perceptions of family relationships. Their findings confirmed the influence of the mentioned variables and also showed the association between self-blame and children’s parentification. Mentioned variables may impact different dimensions of the children’s psychological features.

In other studies, DV had led to negative and overwhelming emotions as well as suppression effect for young children (Thornton 2014) and reduced psychological wellbeing and social support satisfaction (2017). Dargis and Koenigs (2017) found interpersonal and affective features of psychopathy as well as possible later psychopathic behaviors in children exposed to DV.

Later in 2018, Cho (2018) reported aggressive behavior, depression, anxiety, and juvenile delinquency for children who had been the witness of domestic violence in their families. In one study on 907 boys and girls exposed to DV, Forke et al. (2018) found higher perpetration for boys and higher combined victimization/perpetration for girls indicating major psychological adverse effects for children exposed to DV. Such psychological problems can alter behavioral developments and decline individuals’ life satisfaction.

Paul (2019) et al. reported post-traumatic stress symptoms in children witness of DV, and Hussain et al. (2019) reported mental depression, humiliation and public ridicule, negative effects on children’s cognitive growth. Cho (2019) reported depression and anxiety, delinquent behavior, affected academic performance, and sociality in South Korean children, and Fogarty et al. (2020) detected emotional-behavioral resilience in children exposed to DV. It can be expected that the future life of children exposed to DV can be influenced by possible effects and major consequences of DV. Mentioned mental and psychological consequences may be long-lasting and affect the children’s personal, social, and educational performance and their life quality (Table 2).

**Table 2:** Included studies evaluating the psychological impacts of domestic violence on children

**Authors (Ref.)**	Year	Country	Sample size	Findings
Levendosky and Bermann (2001)	2001	USA	120	Ecological framework and trauma theory for the effects of DV on children. Altered behavior in developing children
Huth-Bocks et al. (2001)	2001	USA	100	Poorer verbal and intellectual abilities
Levendosky et al. (2002)	2002	USA	62	Trauma symptoms as common reactions in children who witness DV. Recommendation for clinicians and school counselors to screen DV in children with behavioral and emotional problems
Spilsbury et al. (2008)	2008	USA	175	Psychological maladjustment, internalizing, and externalizing problems
Fusco and Fantuzzo (2009)	2009	USA	1581	Children involved in DV events physically and emotionally
Torteya et al. (2009)	2009	USA	190	DV was associated with internalizing or externalizing symptoms. Influence on children’s adaptation
Rigterink et al. (2010)	2010	USA	130	Increased baseline vagal tone, stress symptoms
Fortin et al. (2011)	2011	Canada	79	Effects on children’s adjustment, internalizing problems, perceived threat, parentification and loyalty conflicts
Thornton (2014)	2014	UK	8	Negative and overwhelming emotions for young children
Naughton et al. (2017)	2017	Ireland	465	Reduced psychological wellbeing and social support satisfaction, the suppression effect
Dargis and Koenigs (2017)	2017	USA	127	Interpersonal/affective features of psychopathy, later psychopathic traits
Cho (2018)	2018	South Korea	1335	Aggressive behavior, depression, anxiety, juvenile delinquency
Forke et al. (2018)	2018	USA	907	Higher perpetration for boys and higher combined victimization/perpetration for girls
Paul (2019)	2019	France	46	Post-traumatic stress symptoms
Hussain et al. (2019)	2019	Pakistan	269	Mental depression, humiliation, and public ridicule, negative effects on children’s cognitive growth
Cho (2019)	2019	South Korea	421	Depression and anxiety, delinquent behavior, affected sociality, and educational performance
Fogarty et al. (2020)	2020	Australia	1060	Emotional-behavioral resilience

## Discussion

Domestic violence has been reported to be expressed in different forms of physical, psychological, and sexual (Shayan et al. 2016, Dargis and Koenigs 2017). Various studies have revealed consequences of DV and concerns about the victims of DV have been being raised for health policymakers (Dargis and Koenigs 2017, Gholamzadeh et al. 2018, Maji 2018). Victims of DV may suffer various physical or mental health complications (Hegarty 2011, Heron and Eisma 2021). Reported psychological complications are depression, anxiety, post-traumatic stress disorder (PTSD), lower self-esteem, increased risk of substance abuse, self-injury, and even suicide (Banerjee et al. 2019, van Der Put et al. 2019). So far, most of the researches has focused on first and direct victims of DV which may be wives or husbands (Drijber et al. 2013). However, recent investigations have been conducted about the effects of DV on indirect victims of DV who are mainly children (Walters 2018). Domestic violence can cause various negative psychological, behavioral, cognitive, and emotional impacts on children (Callaghan et al. 2018, Forke et al. 2019).

DV may cause direct impacts on victims such as homicide deaths and living with poor health and declined earnings (Rawlings and Siddique 2020). Empirical studies have studied the relationship between domestic violence experienced by mothers and health outcomes of their children, and it has been found that children may suffer negative early and delayed effects (Yount, et al. 2011).

Our systematic survey showed that most of the children who witness DV may display psychological symptoms such as internalizing, externalizing behaviors, and depression (Martinez‐Torteya et al. 2009, Fortin et al. 2011). As the social activities of children are impacted by the mentioned mental and psychological problems, DV may cause decline in social activity of children who are victims or witnesses of DV (Howarth et al. 2019, Wahyuni et al. 2019). Such children may have poor school and educational performance that may be due to affected verbal and intellectual abilities (Huth-Bocks et al. 2001). Trauma related symptoms are also common reactions in children who witness DV (Levendosky et al. 2002).

The children witness DV may be less pleased to have participated in social activities. So, clinicians and school counselors are recommended to monitor and care about DV in children which may be presented by behavioral and emotional changes. The children exposed to DV also may show to have lower IQ levels (Koenen et al. 2003). Post-traumatic stress disorder (PTSD) was another reported consequences of DV in children witnesses of domestic violence (Rigterink et al. 2010, Paul 2019). It seems that children experience a trauma like event when are witness or exposed to DV, wheather the exposure is direct or indirect. Besides, reduced psychological wellbeing and social support satisfaction, suppression effect are reported as health consequences of DV witnessing in children (Naughton et al. 2017, Maji 2018).

Children exposed to DV may show anxiety, aggressive behavior, juvenile delinquency, interpersonal, and affective features of psychopathy as well as later psychopathic traits (Dargis and Koenigs 2017, Cho 2018, Maji 2018). Furthermore, the child witnessing domestic violence can reveal far-reaching consequences for children (Yakob 2018). Mental depression, humiliation, and public ridicule, negative effects on children’s cognitive growth (Ahmadzad-Asl et al. 2016, Hussain et al. 2019, Tonsing et al. 2020) as well as emotional-behavioral resilience (Fogarty et al. 2019, Fogarty et al. 2020).

Children witness of DV have reported to be depressed fearful and inhibited. Besides, they may suffer internalizing and behavioral disorders, antisocial and aggressive behaviors (Gomma et al. 2019). Moreover, children exposed to DV may also suffer physical complications such as general pain and aches. Bedwetting, irritable and irregular bowel disease (IBD) and cold sores may be other complain of such children (Turhan 2022). Children may also become nervous with signs of fatigue. Such children may have poor personal hygiene and an increased desire to be involved in high-risk games, self-abuse, and suicide (Sureka and Kesarwani 2022).

Children exposed to DV suffer short‐ and long‐term behavioral and mental health impacts. As one of the main human resources of society, any maltreatment such as DV can cause major behavioral and antisocial consequences in growing and developing children. Several studies had paid to interventions for treatment and minimizing the psychological consequences of domestic violence in preschool, school, and young children, which were not included in our systematic review. Our study included studies in which the mental, behavioral, and psychological consequences of DV had been evaluated in witnessing children.

## Strengths and limitations

**Fig. 1:**
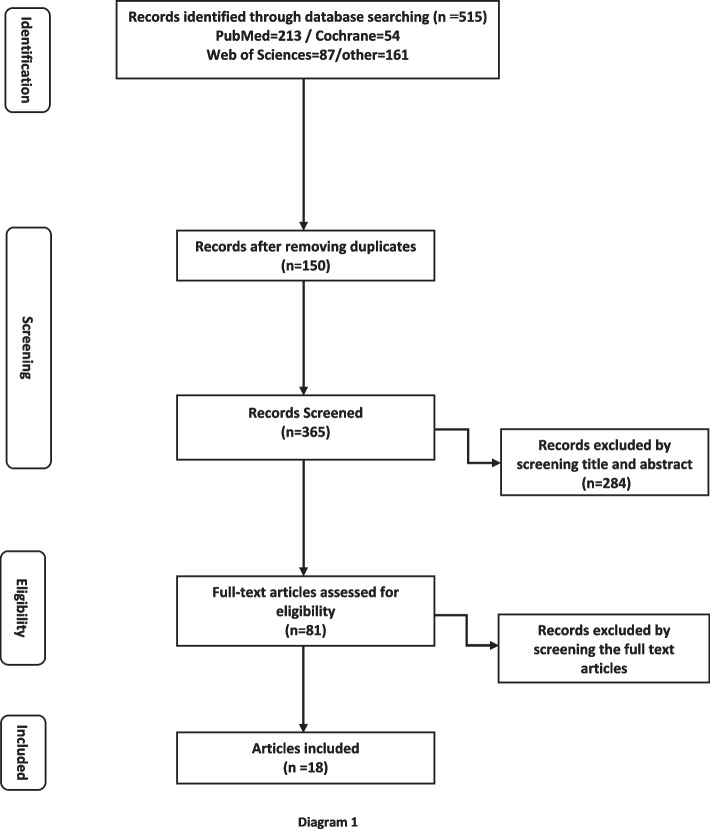
The strategy of searching articles based on Preferred Reporting Items for Systematic Reviews and Meta-Analyses (PRISMA)

In this systematic review, we tried to reduce bias on the available evidence of the psychological consequences of DV on witnessing children. A careful research design and comprehensive sampling procedures was applied for finding related literature. However, data collection method might lead to sampling bias, and we may have missed some studies that fulfill the inclusion criteria. Long term duration range of the studies involved in our systematic review was one of the strong points. Besides, we only included original research studies found by application of MeSH keywords and we did not evaluate the systematic reviews, narrative reviews nor meta-analysis studies. Moreover, we identified the relevant studies in an independently duplicated manner (Fig. 1).

## Conclusions

The findings of the present study reveal that children exposed to domestic violence suffer various psychological, mental, and behavioral complications which may be short and long-lasting. Children exposed to DV may deal with declined educational performance and social abilities. Psychological complications may be internalization problems, depression, post-traumatic stress disorders, and externalization symptoms such as aggressive behaviors and even lower levels of IQ. Educational programs for parents as well as trained school caregivers and health policymakers can diminish and prevent the complications of DV on witnessing children. Precise psychological investigations and standard psychological tests may be helpful for assessment of intellectual ability (or intelligence), academic skills (or achievement), cognitive functions such as memory, focus and attention, and visual-motor coordination, neurocognitive tests, personality tests, and brain dominance. Considering the importance of children mental hygiene, health policymakers should consider facilities to detect children with signs of maltreatment and exposed to domestic violence. In this regard, trained teachers or school counselors will be helpful for screening and early detection of such children. So, social supports and therapies may be more effective by early detection affairs.

## Data Availability

All data generated or analyzed during this study are included in this published article.

## Abbreviations

DV: Domestic violence
IQ: Intelligence quotient
VT: Vagal tone
PTSD: Post-traumatic stress disorder
